# Development of an Electrochemical Reactor Prototype for Biodiesel Production Powered by Photovoltaic Energy

**DOI:** 10.1021/acsomega.6c07137

**Published:** 2026-09-11

**Authors:** Gabriel Felipe Rohling, Josiane de Almeida Freire, Katharine Margaritha Satiro Braz, Fernanda Oliveira Lima, André Lazarin Gallina, Thiago Anjos, Ricardo F. Schumacher, Letiére Cabreira Soares

**Affiliations:** † 232192 Universidade Federal da Fronteira Sul, Campus Realeza, Paraná 85770-000, Brazil; ‡ 28133 Universidade de São Paulo, Campus Butantã, São Paulo 05508-010, Brazil; § Universidade Estadual do Centro Oeste, Campus Cedeteg, Paraná 85040-167, Brazil; ∥ 28118 Universidade Federal de Santa Maria, Santa Maria, Rio Grande do Sul 97105-900, Brazil

## Abstract

The continuous increase in global energy demand has intensified the search for renewable alternatives capable of replacing fossil fuels. In this study, an electrochemical reactor prototype powered by photovoltaic energy was developed for biodiesel production from sunflower oil, using 304 stainless steel electrodes. The system was designed to operate under mild conditions, with adjustable voltage (10–30 V) and different oil:methanol molar ratios (1:3–1:9). The influence of these parameters on the physicochemical properties of the produced biodiesel was evaluated, including acid value, ester content, yield, and density. The electrochemical process achieved yields above 90% and ester contents exceeding 96.5%, meeting the standards established by ANP and EN 14214. The acid value decreased from 1.85 mg KOH·g^–1^ in the feedstock oil to 0.18–0.54 mg KOH·g^–1^ in the biodiesel, indicating efficient conversion of free fatty acids. The density of the samples (881–889 kg·m^–3^) remained within the limits recommended by regulatory agencies. Statistical analysis (Welch’s ANOVA) confirmed significant effects of the molar ratio and applied potential on the final acid value of the biodiesel. The results demonstrate the technical feasibility of integrating photovoltaic energy with electrolysis for sustainable biodiesel synthesis, highlighting the potential of low-cost materials and renewable energy sources for biofuel production.

## Graphical abstract

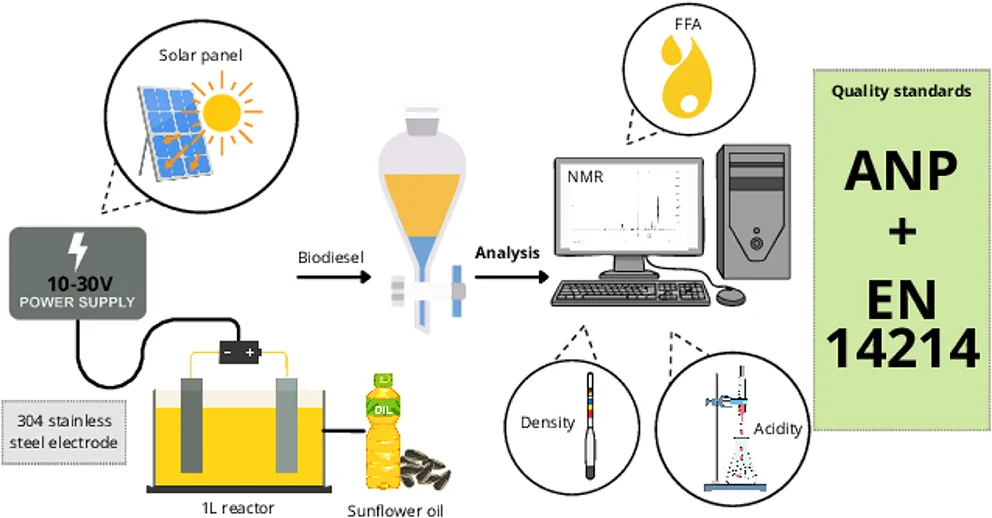

## 1 Introduction

Global energy consumption and, consequently, the demand for energy production have steadily increased over the past decades.^1^ This growth has been predominantly supplied by nonrenewable energy sources, such as fossil fuels, which still form the basis of the global energy matrix, accounting for approximately 80% of worldwide energy consumption.^2^ However, this dependence poses significant challenges, including greenhouse gas emissions, energy insecurity, and environmental impacts, making the search for alternative and renewable energy sources increasingly urgent.^3^

In this context, biodiesel has emerged as a promising alternative, offering advantages such as high biodegradability, reduced pollutant emissions, and a more balanced carbon cycle.^4^ Chemically, biodiesel consists of a mixture of fatty acid alkyl esters, primarily produced via transesterification of oils or fats with an alcohol, typically catalyzed by homogeneous catalysts.^5^ Nevertheless, homogeneous catalysis presents several limitations, including the requirement for feedstocks with low acidity and moisture content, soap formation, difficulties in biodiesel separation, and the generation of aqueous effluents, all of which increase production costs.^6^

As an alternative, electrochemical methods have been investigated, offering advantages such as greater tolerance to free fatty acids and water, thereby enabling the use of low-quality residual oils. Furthermore, this approach can operate under mild conditions, reducing reaction time and energy consumption compared to conventional routes.^7^

The first study demonstrating the feasibility of the electrochemical route was conducted by Guan and Kusakabe (2009), who produced biodiesel from corn oil and waste oil with yields exceeding 97%, even in the presence of 2% water.^8^ More recent studies have continued to advance this field. Liu et al. (2024) employed a magnetic catalyst (CeO_2_/ZSM-5@Fe_3_O_4_) in combination with platinum electrodes, achieving a 95% yield at room temperature.^9^ Putra et al. (2016) used chitosan in gel form (hydrogel and xerogel) as a heterogeneous catalyst in electrolysis with graphite electrodes, obtaining a 99.4% yield in only 30 min.^10^

Similarly, Asl et al. (2020) produced biodiesel from waste cooking oil using graphite electrodes and a homogeneous catalyst (NaOH), reaching a 98% yield within 2 h, with the advantage of no voltage drop or temperature variation during the process.^11^ Complementarily, Karimi and Saidi (2022) optimized biodiesel production from neem oil (*Azadirachta indica*) via electrolysis using H_2_SO_4_ as an acid catalyst and response surface methodology (RSM), achieving an 80% yield under ambient conditions and demonstrating the feasibility of non-edible feedstocks using this method.^12^ Some more representative studies on electrochemical biodiesel production are summarized in Table 1. The electrochemical production of biodiesel relies on the *in-situ* generation of methoxide ions through the electrolysis of the reaction medium. At the cathode, water is reduced to hydroxide ions, which react with methanol to form methoxide ions. At the anode, oxidation reactions such as chlorine or oxygen evolution may occur depending on the electrolyte composition. The methoxide ions attack the carbonyl group of triglycerides (TG), yielding biodiesel and glycerol. A schematic representation of this reaction is shown in Figure 1, as reported by Asl et al. (2020).^11^

**Figure 1:**
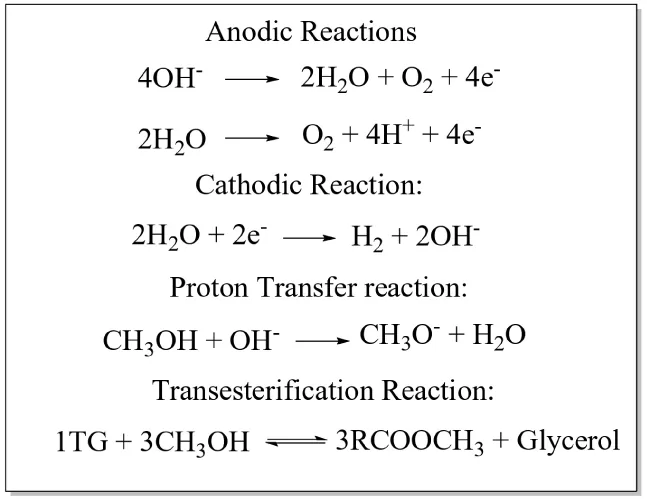
Electrochemical reactions and transesterification.

**Table 1:** Literature Survey on Electrochemical Biodiesel Synthesis

Reference	Reactor design	Electrodes	Feedstock	Operate conditions	Biodiesel yield (%)	Energy consumption (kWh/kg)	Main advantages
This study	Batch electrochemical reactor	Stainless steel	Sunflower oil	KOH; 10–30 V, 1:3–1:9 (oil:MeOH); 25 °C, 60 min, 2% H_2_O	>90	7.20 × 10^–3^	Photovoltaic-powered; low energy consumption, low-cost electrodes and corrosion-resistant
Karimi and Saidi (2024)^13^	Semi-batch glass reactor	Graphite	Neem oil	Mordenite-sulfated catalyst; 10 V, 1:10 (oil:MeOH), THF, NaCl, 25 °C, 60 min, 2% H_2_O	85	-	High yield; non-edible feedstock and good catalyst reusability
Habibzadehnesami et al. (2026)^14^	Electrochemical microenvironment	Nickel	Frying oil	3D Ti-MOF@ZnAl-LDH;18–20 V; 1:6 (oil:MeOH); NaCl; 25 °C; 100 min	98.4	-	Simultaneous transesterification/esterification and mild operating conditions
Söyler et al. (2026)^15^	Batch electrochemical reactor	Graphite and titanium	Waste cooking oil	KOH; 20.3–60 V; 45 wt % MeOH; Acetone; 35–50 °C; 46.22-48.18 min	83.45–93	0.25–1.19	High biodiesel yield, short reaction time and low energy consumption
Khan et al. (2026)^16^	Batch electrochemical cell	Graphite and Nickel-coated graphite	Waste cooking oil	30 V; 1:25 (oil:MeOH);NaCl; 25 °C; 120–150 min; 0.5% H_2_O	96.8	1.22	Ultra-low cost, high operational stability and high electroactive area

Despite the promising results summarized in Table 1 and the reactions shown in Figure 1, the energetic and economic feasibility of the process remains a challenge. Most studies still rely on conventional electrical energy for electrolysis, often derived from nonrenewable sources. Moreover, the use of materials such as platinum electrodes, specialized reagents, and catalysts increases operational costs, hindering industrial scalability.

In this context, the present study proposes the development of an electrochemical reactor for biodiesel production powered by photovoltaic energy, employing accessible and durable materials such as 304 stainless steel electrodes. Additionally, reaction parameters influencing biodiesel quality were evaluated, including acid value, density, ester content, and final yield.

## 2 Materials and Methods

### 2.1 Materials

The feedstock used in this study was sunflower oil donated by the Brazilian Federal Revenue Service, originating from a seizure at the border between Brazil and Argentina. Prior to its use, a preliminary analysis of its physicochemical parameters was conducted. The analytical-grade reagents employed for sample analysis and preparation of the reaction mixtures were sodium hydroxide (NaOH), potassium hydroxide (KOH), ethanol (99.8% purity), diethyl ether (98% purity), methanol (99.9% purity), deuterated chloroform (99.8% purity), and tetramethylsilane (TMS).

### 2.2 Assembly of the Electrochemical Reactor Prototype

The reactor prototype was constructed using a cylindrical-conical vessel with a total capacity of 1 L. The system was powered by photovoltaic energy, consisting of an 80 W solar panel, an inverter, a power supply with voltage and current control, a 12 V battery, and a 12 V electric pump with a flow rate of 1.5–2 L·min^–1^, responsible for homogenizing the reaction mixture. Additionally, a switching system was implemented to allow operation using the conventional electrical grid, ensuring continuous operation in the absence of solar radiation. The photovoltaic panel was used to charge the battery, whereas the electrochemical reactor was supplied by the regulated DC power source. This configuration ensured that the applied potential difference remained constant throughout the experiments, regardless of short-term fluctuations in solar irradiance.

The reactor was equipped with a pair of 304 stainless steel electrodes, each with a surface area of 50 cm^2^ and separated by a distance of 1 cm. This material, composed of an austenitic alloy containing 18–20% chromium and 8–10% nickel, with the balance being iron,^17^ exhibits high corrosion resistance and lower cost, characteristics that make it suitable for the electrolysis process. A schematic illustration of the reactor system is presented in Figure 2.

**Figure 2:**
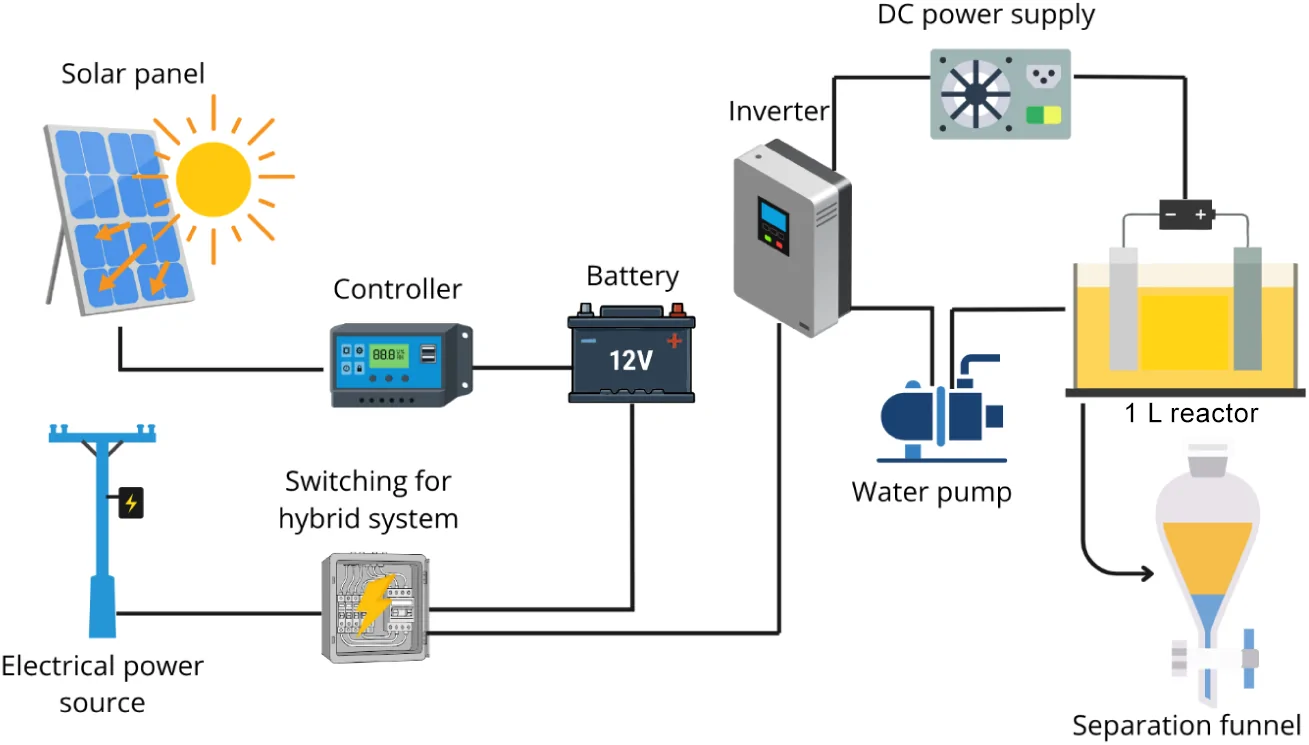
Schematic illustration of reactor system.

### 2.3 Biodiesel Synthesis by Electrolysis

For biodiesel synthesis, the reactor was charged with 700 mL reaction mixture containing methanol, sunflower oil, potassium hydroxide, and distilled water. The oil:methanol molar ratios investigated were 1:3, 1:3.5, 1:4, 1:6, 1:7, and 1:9. The concentration of potassium hydroxide was fixed at 1 wt % relative to the mass of the oil, and 2 wt % of distilled water was added based on the total mixture mass.

The electrolysis reaction was carried out on the reactor for 60 min at room temperature under constant stirring, applying a constant potential difference (PD). The PD was adjusted to 10, 20, or 30 V depending on the experimental condition.

The Specific Energy Consumption (SEC) of the process was calculated to evaluate the energy efficiency of the electrochemical system. The SEC (in kWh per kg of biodiesel) was determined considering the total electrical energy consumed during the reaction and the mass of biodiesel produced, according to the methodology described by Söyler et al. (2026). The total energy consumption was calculated as the sum of the energy demanded by the electrolysis process and the stirring system. The energy consumed of the optimized condition during electrolysis was determined using eq 1, where V is the applied potential difference (V), I is the electric current (A), t is the reaction time (h), and m is the mass of biodiesel obtained.

1 $$\mathrm{SEC}=\frac{V\times I\times t}{1000\times m}$$

The electric current was measured during the electrolysis process using a digital multimeter model ICEL MD-6199.After completion of the reaction, the mixture was transferred to a separatory funnel to allow glycerol decantation. The resulting biodiesel phase was divided into two portions. The first portion was washed with distilled water (30% of the biodiesel volume) and subsequently dried in an oven at 105 °C for 2 h. The second portion was directly subjected to the drying process under the same conditions, without a washing step. This methodology was adapted from Asl et al. (2020).^11^

### 2.4 Physicochemical Characterization of Biodiesel

The physicochemical characterization included the determination of the initial acid value (AV), free fatty acid (FFA) content, and density of the sunflower oil, as these parameters are essential for assessing the initial quality of the feedstock and for subsequent comparison with the biodiesel produced in the reactor.

The oil density was determined in kg·m^–3^ using a digital densimeter (Anton Paar, model DMA 35). The acid value and free fatty acid content were determined according to the titrimetric method described by Lüneburger et al. (2022).^18,19^ In this procedure, a 0.01 mol·L^–1^ NaOH solution was used as the titrant, while the analyte consisted of 2 g of sample dissolved in 25 mL of a diethyl ether:ethanol mixture (2:1, v/v), using phenolphtalein as the acid–base indicator.

The acid value (mg KOH·g^–1^) and the free fatty acid content, expressed as percentage of oleic acid, were calculated according to eqs 2 and 3, respectively, where *v* represents the volume of NaOH used, *f* is the correction factor of the titrant solution, and *p* is the sample mass in grams.

2 $$AV=\frac{v\times f\times 56.1}{p}$$

3 $$FFA=\frac{\left(v\times \left(f\times M\right)\times 28.2\right)}{p}$$

### 2.5 Determination of Ester Content

The ester content confirms and quantifies the conversion of triglycerides into biodiesel. The ester content of the biodiesel samples was determined by nuclear magnetic resonance spectroscopy of hydrogen (^1^H NMR) using a Bruker DPX 400 spectrometer operating at 400 MHz for ^1^H. Samples were dissolved in deuterated chloroform (CDCl_3_), with TMS used as the internal standard. The ester content was calculated based on the integration values of the methoxy group (−OCH_3_) at chemical shift (δ) of 3.6 ppm and the α-carbonyl methylene (−CH_2_−), (δ) = 2.3 ppm and eq 4.

4 $$EC\left(\%\right)=\left[\left(\frac{Int.O{CH}_{3}}{3}\right)/\left(\frac{Int.O{CH}_{2}}{2}\right)\times 100\right]$$

Where EC is the conversion to methyl esters in %, Int. OCH_3_ corresponds to the integration of the hydrogens from the methoxy group and Int. CH_2_ is the hydrogens from α-carbonyl methylene integration signal.

### 2.6 Scanning Electron Microscopy

The surface morphology of the stainless-steel electrodes was evaluated before and after the electrolysis experiments using scanning electron microscopy (SEM) (VEGA4 LM, TESCAN). Prior to analysis, the electrodes were rinsed with distilled water to remove residual reaction products, dried at room temperature, and analyzed without further surface treatment. The SEM micrographs were obtained to assess possible morphological changes associated with the electrolysis process, including the presence of corrosion, pitting, cracks, or other surface degradation features.

### 2.7 Statistical Analysis

Statistical analyses were performed using JASP software (version 0.19.3.0, 2025). Levene’s test for homogeneity of variances indicated a violation of the homoscedasticity assumption (*p* <0.05). Therefore, Welch’s analysis of variance (Welch’s ANOVA) was applied to evaluate the effects of the independent variables (molar ratio and applied potential difference) on the acid value. Post-hoc comparisons were conducted using Holm’s correction method, considering a significance level of 95% (*α* = 0.05).

## 3 Results and Discussion

### 3.1 Physicochemical Characterization of Sunflower Oil

Sunflower oil was evaluated as a feedstock for biodiesel production via electrolysis, with emphasis on the effects of molar ratio and applied PD on the electrochemical process.

The physicochemical characterization of the sunflower oil was performed prior to the reaction, and the properties obtained are summarized in Table 2.

**Table 2:** Physicochemical Properties of Sunflower Oil Used for Biodiesel Production

Parameter	Unit	Value
Acid value	mg KOH·g^–1^	1.85 ± 0.07
Free fatty acids	%	0.93
Density	kg·m^–3^	911.0

According to Table 1, the sunflower oil exhibited an acid value, free fatty acid content, and density of 1.85 mg KOH·g^–1^, 0.93%, and 911.0 kg·m^–3^, respectively. These results indicate that oil presents a higher acidity compared to values reported in the literature. For instance, Ebrahimian et al. (2022)^20^ reported an AV of 0.9 mg KOH·g^–1^ for the same lipid matrix.

Several authors have stated that, in order to achieve high yield in biodiesel production via homogenous alkaline catalysis, oils with an acidity below 1 mg KOH·g^–1^ and FFA content lower than 0.5% are required to prevent catalyst consumption and soap formation.^21,22^ In this context, the use of the electrochemical transesterification method may significantly enhance reaction performance by favoring triglyceride conversion into esters while minimizing the formation of undesirable by-products. Regarding density, the value obtained in this study is comparable to that reported by Mora et al. (2024),^23^ who found a density of 924.9 kg·m^–3^ for sunflower oil.

### 3.2 Biodiesel Yield and Ester Content

In the electrochemical transesterification process, hydroxide ions are generated at the cathode and react with the methanol to form methoxide ions, which act as nucleophiles by attacking the carbonyl carbon of triglycerides, thereby initiating the formation of methyl esters.^11^ Figure 3 presents: (a) the biodiesel yield obtained under different reaction conditions and (b) the ester content determined by ^1^H NMR spectroscopy.

**Figure 3:**
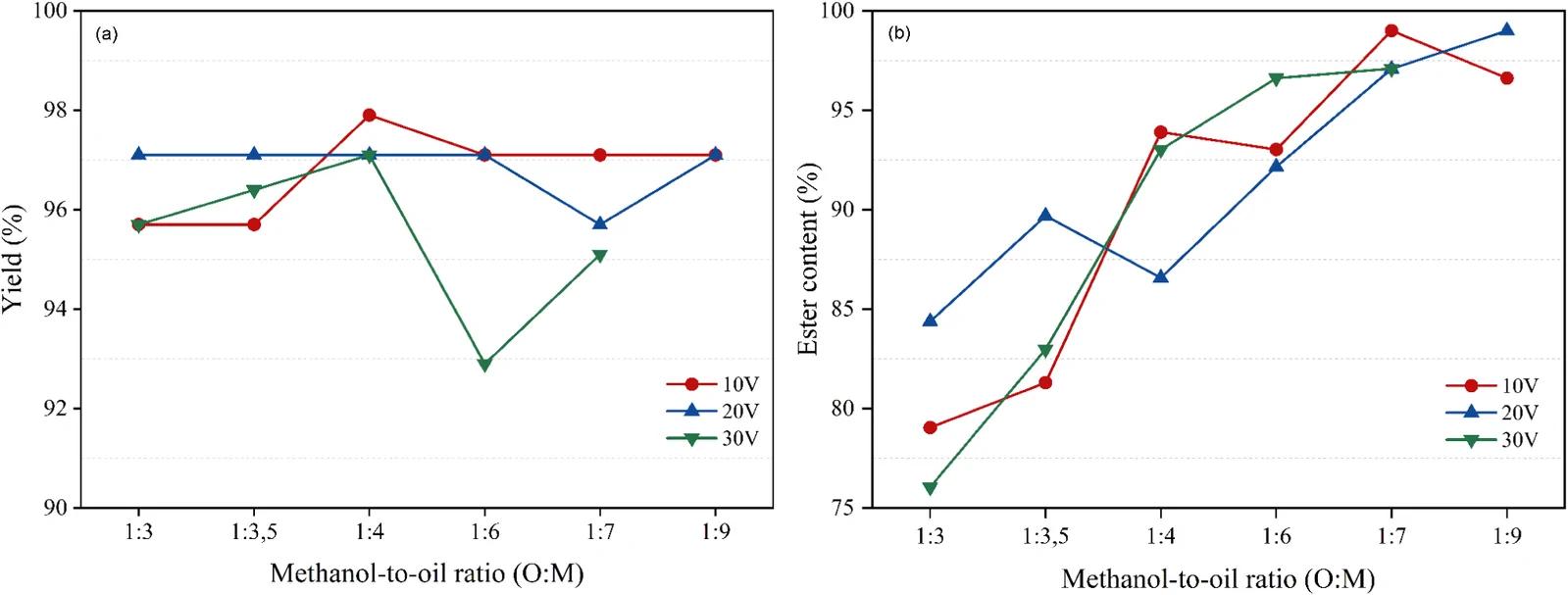
Transesterification reaction yield and ester content of biodiesel; (a) biodiesel yield; (b) biodiesel FAME.

The reaction yield (Figure 3a) exceeded 90% under all tested conditions, except at the 1:9 oil-to-methanol molar ratio under an applied PD of 30 V. Under these conditions, the system became excessively conductive, leading to electrical failure and preventing completion of the experiment. These results indicate performance comparable to that reported by Guan and Kusakabe (2009)^8^ and Irawan et al. (2019),^24^ who achieved reaction efficiencies above 90% in similar electrochemical systems.

As shown in Figure 3b, despite the high reaction yields, the conversion to alkyl esters (NMR spectra available in supplementary material) did not reach the minimum limit established by the National Agency of Petroleum, Natural gas and Biofuels (ANP) and the EN14214 standard (≥96.5%) under certain conditions, particularly at lower oil-to-methanol molar ratios (1:3, 1:3.5, and 1:4), regardless of the applied PD. This limitation may be associated with the chemical equilibrium of the transesterification reaction, which is reversible and tends to shift toward the reactants at lower alcohol proportions.

According to Al-Haimi et al. (2025),^25^ increasing the methanol concentration shifts the chemical equilibrium toward the products, in accordance with Le Chatelier’s principle, thereby enhancing triglyceride conversion into biodiesel. Thus, at lower molar ratios, the reaction does not reach complete conversion, resulting in reduced ester content and the presence of residual mono-, di-, or tri- glycerides. Conversely, increasing the oil-to-methanol molar ratio to 1:6, 1:7, and 1:9 shifts the equilibrium toward ester formation, leading to ester contents above 96.5%, except under the 1:6 condition at 10 and 20 V (93.02% and 92.16%, respectively). Under these conditions, the lower applied potential may not have provided sufficient energy to efficiently promote water reduction and, consequently, hydroxide ion generation, as described by the electrochemical reactions shown in Figure 1.

Since these ions participate in proton transfer reactions with methanol to form methoxide ions, which catalyze the transesterification process, a lower production of hydroxide ions limits the availability of these reactive species in the reaction medium. Consequently, this may reduce catalytic efficiency and potentially allow competing side reactions to occur, thereby decreasing the overall conversion efficiency.

### 3.3 Free Fatty Acid Content of the Produced Biodiesel

The results of the present study demonstrated that the use of an electrochemical reactor for biodiesel synthesis can significantly reduce the free fatty acid (FFA) content in the final product. This effect is illustrated in Figure 4, which presents the FFA results for biodiesel samples (a) before washing and (b) after the washing process.

**Figure 4:**
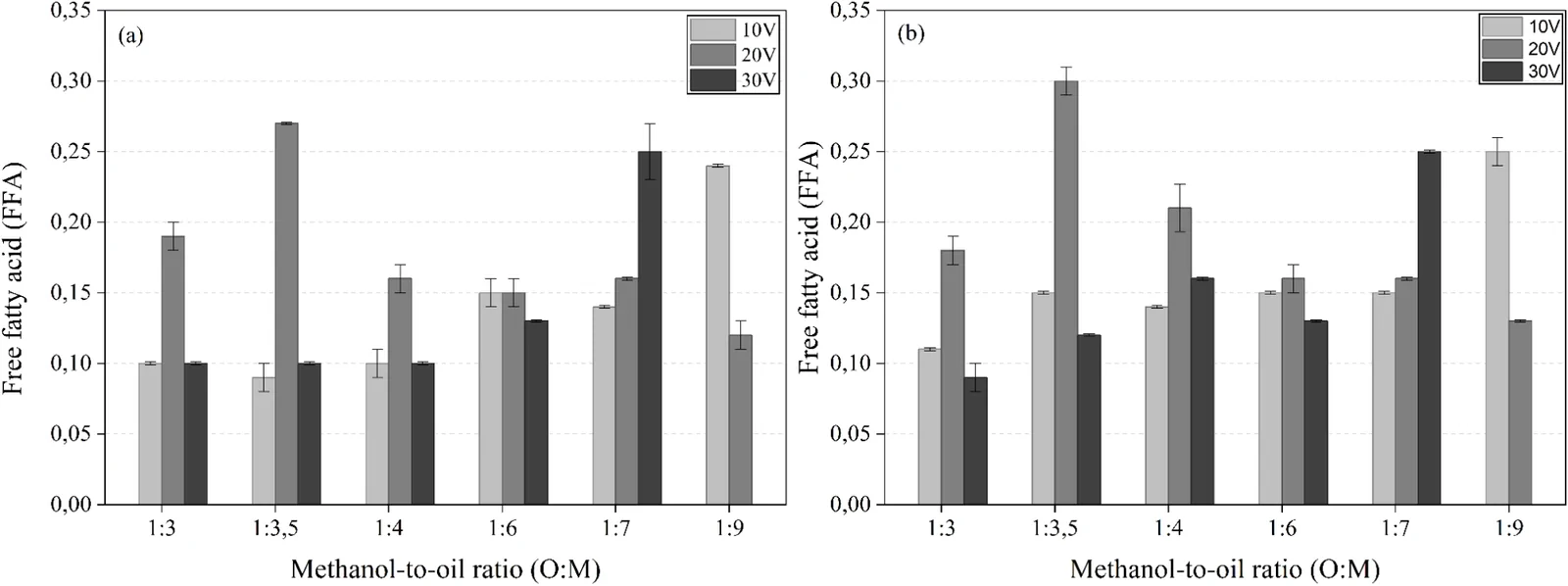
Influence of molar ratio and applied PD on free fatty acid content; (a) before washing; (b) after washing process.

According to Figure 4, the FFA values ranged from 0.09% to 0.27% for the unwashed biodiesel and from 0.09% to 0.30% for the washed biodiesel, demonstrating a substantial reduction compared to the initial FFA content of the sunflower oil (0.93%). These results indicate that the electrochemical process contributes to the conversion of free fatty acids into esters, thereby reducing the acidity of the produced biodiesel. Similar findings were reported by Darwin et al. (2023),^7^ who also observed a significant decrease in FFA content in electrochemically assisted transesterification systems.

In general, it was observed that higher molar ratios resulted in increased FFA contents when potentials of 10 and 30 V were applied. Conversely, the highest FFA values at lower molar ratios (1:3, 1:3.5, and 1:4) were obtained under 20 V (Figure 4a and b). This behavior may be associated with the balance between transesterification and triglyceride hydrolysis reactions, which are influenced by both the applied potential and the oil:methanol molar ratio. The applied PD directly affects the generation rate of hydroxide ions, which are essential for methoxide formation and subsequent transesterification. However, under non-optimized conditions, such as excessive methanol or inadequate potential, the reaction environment may favor competing pathways.

In the study by Liu et al. (2024),^9^ using CeO_2_/ZSM-5@Fe_3_O_4_ heterogeneous catalyst, the authors demonstrated that the applied potential plays a crucial role in hydroxide ion generation during electro-assisted systems. They reported that deviations from optimal conditions, including excess methanol or inappropriate voltage, reduced biodiesel yield and promoted side reaction such as hydrolysis, leading to increased FFA formation. A similar mechanistic interpretation can be proposed for the present system.

### 3.4 Acid Value of the Produced Biodiesel

The results of the present study showed that the acid value of the unwashed biodiesel (0.18–0.54 mg KOH·g^–1^) was slightly lower than that of the washed biodiesel (0.18–0.60 mg KOH·g^–1^) across evaluated conditions. Biodiesel washing is an essential step for the removal of residual impurities, such as traces of catalyst, soaps, glycerol, and methanol, which may affect the quality of the final product. The increase in acid value after the washing process can be attributed to the elimination of residual basic catalyst, which may have influenced the acidity measurement in the unwashed biodiesel. Therefore, the washing step is essential to minimize impurities and contamination.

In addition, the results of the present study also demonstrated that the transesterification process via electrolysis can significantly reduce the acid value of the initial oil (1.85 mg KOH g^–1^) when compared to the final biodiesel (Figure 5a and b). These results are similar to the study by Annal et al. (2024),^26^ who reported a reduction from 14.25 mg KOH·g^–1^ to 0.5 mg KOH·g^–1^ in the final biodiesel produced by electrolysis.

**Figure 5:**
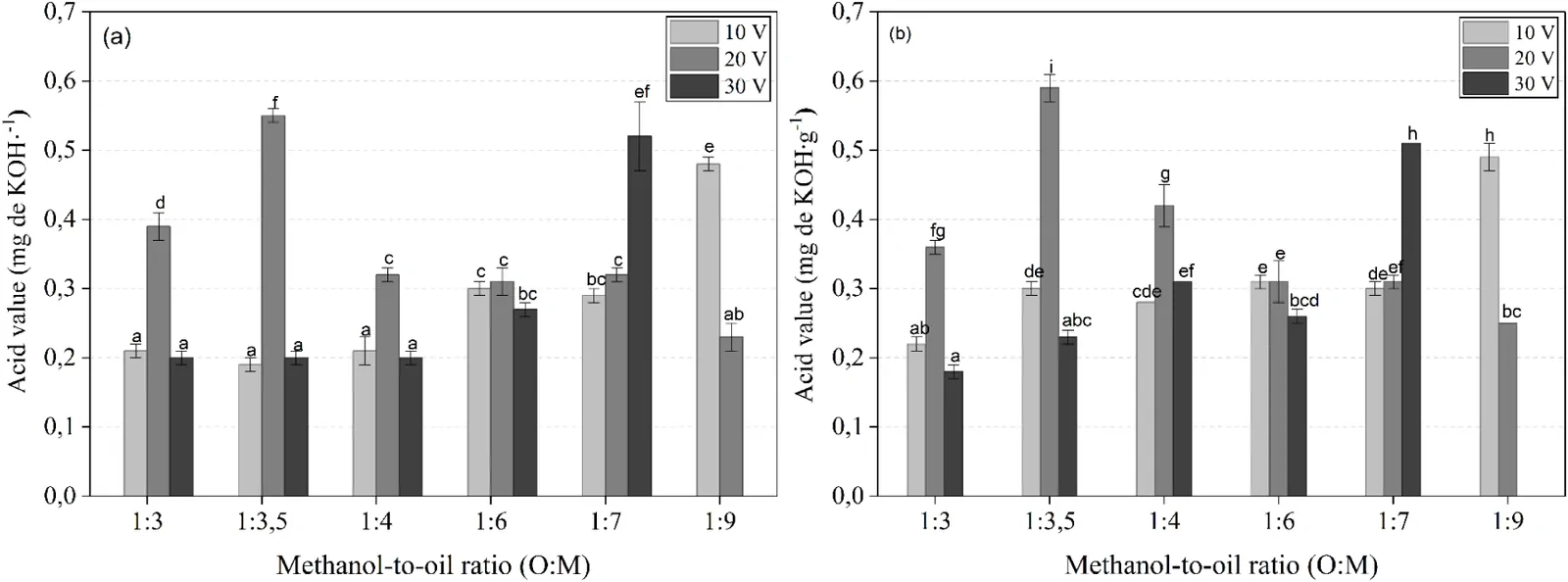
Influence of molar ratio and applied PD on the acidity index of biodiesel; (a) before washing; (b) after washing process; Identical letters do not differ statistically from each other (*p* > 0.05).

As shown in Figure 5b, when potential differences of 10 and 30 V were applied, increasing the molar ratio led to a progressive increase in the acid value, ranging from 0.22 to 0.49 mg KOH·g^–1^ and from 0.18 to 0.50 mg KOH·g^–1^, respectively. This behavior suggests that the increase in acidity does not depend solely on the magnitude of the applied PD, but rather on the balance between hydroxide ion generation, methoxide ion formation, and the competing reactions occurring in the reaction medium.

At lower PD (10 V), the lower current density reduces the rate of OH^–^ formation and, consequently, the generation of CH_3_O^–^, which may limit triglyceride conversion into biodiesel and favor the occurrence of partial ester hydrolysis. On the other hand, at higher PD (30 V), water electrolysis is intensified, resulting in greater OH^–^ production and, consequently, increased formation of H_2_O molecules in the system through the reaction with methanol, which may also lead to an increase in the acid value due to the formation of free fatty acids.^11–27^

Conversely, the 20 V condition exhibited a distinct behavior, in which the acidity decreased with increasing molar ratio. This result suggests that, within this intermediate potential range, sufficient hydroxide ions are generated to promote the transesterification reaction without causing excessive hydrolysis. The increase in methanol molar ratio shifts the chemical equilibrium toward the formation of methyl esters,^28^ which contributes to the reduction of free fatty acids observed at higher molar ratios, in agreement with the experimental results reported by Ranchman et al. (2018)^29^ and also with Figure 3b, where higher methanol amounts resulted in greater ester conversions.

### 3.5 Biodiesel Density

Biodiesel density is an important quality parameter, as it influences engine performance, fuel atomization, and combustion efficiency.^30^ The results obtained in the present study ranged from 881.0 to 889.9 kg·m^–3^ (Figure 6) and did not show significant differences among the tested variables in terms of the density of the produced biodiesel, indicating that regardless of the applied potential difference or molar ratio used, the density remained unchanged. In addition, other studies have reported similar densities for biodiesel produced by electrocatalysis, such as Xia et al. (2022),^31^ who found a value of 871 kg·m^–3^, and Asl et al. (2020),^11^ who reported a density value of 880 kg·m^–3^ for biodiesel produced via electrolysis.

**Figure 6:**
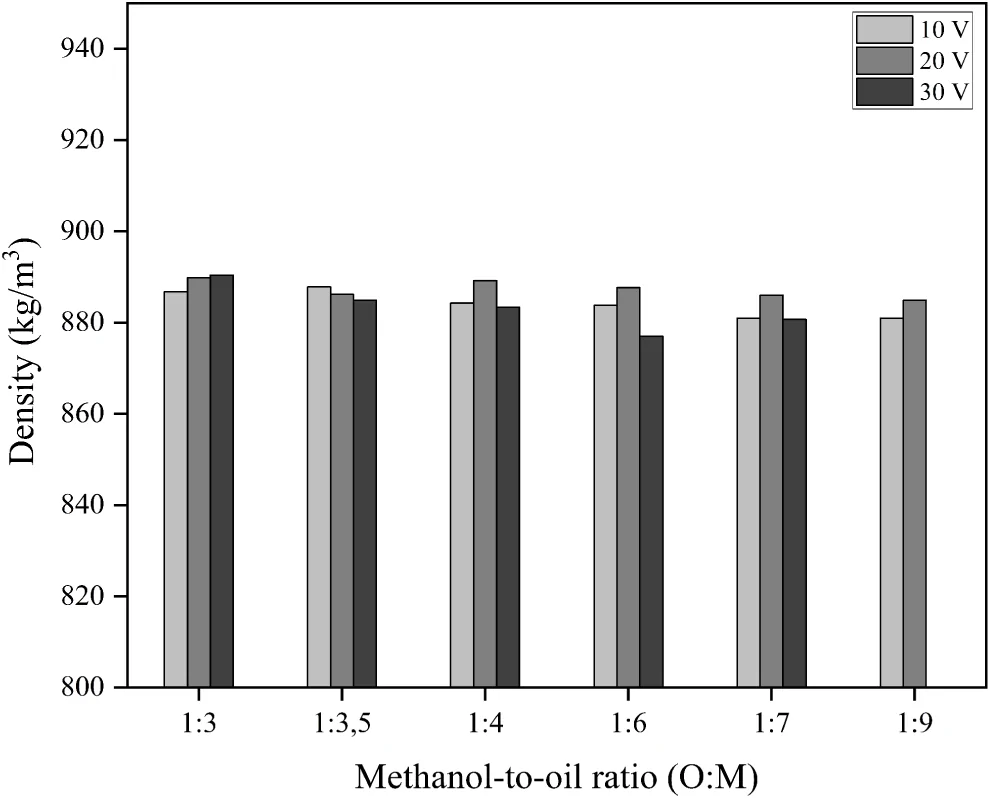
Influence of molar ratio and applied PD on biodiesel density.

### 3.6 Compliance with Biodiesel Quality Standards

The quality of the produced biodiesel was evaluated based on the parameters established by the National Agency of Petroleum, Natural Gas and Biofuels (ANP) and the European standard EN 14214, which define specific limits for properties such as acid value, density, and methyl ester content. According to the ANP and EN 14214 specifications, biodiesel must present an acid value ≤ 0.50 mg KOH·g^–1^, density between 850–900 kg·m^–3^, and ester content ≥ 96.5%.

In the present study, all samples presented density values within the specified range, varying between 881 and 889.9 kg·m^–3^. The acid value was significantly reduced in relation to the initial oil (1.85 mg KOH g^–1^), reaching values between 0.18 and 0.54 mg KOH·g^–1^, which confirms the efficiency of the electrochemical route in converting free fatty acids. The ester content exceeded 96.5% under several experimental conditions, especially at higher molar ratios (1:6 to 1:9).

These results demonstrate that the biodiesel obtained through the electrolysis process met the regulatory quality requirements, presenting properties comparable to those of biofuels produced by conventional catalytic transesterification. Thus, the proposed electrochemical route proves to be technically feasible and compatible with the requirements for application in internal combustion engines, reinforcing its potential as a sustainable alternative to traditional methods.

### 3.7 Favorable Conditions

Based on the experimental results and statistical analysis, the optimized condition identified was a methanol-to-oil molar ratio of 1:7 applied with a potential difference of 20 V. Under this condition, a favorable combination was observed between high ester content (≥96.5%), acid value within regulatory limits (≤0.50 mg KOH g^–1^), and density compatible with ANP and EN 14214 (850–900 kg·m^–3^). In addition, operation at 20 V minimizes the risk of excessive conductivity and electrical failure events recorded under 30 V at higher molar ratios.

### 3.8 Electrode Surface Morphology

The SEM micrographs of stainless-steel electrodes before and after electrolysis are presented in Figure 7. No visible evidence of corrosion, pitting, cracks, or other significant surface degradation was observed after the use of the electrodes in all electrochemical tests conducted in this study. The surface morphology remained comparable to that of the unused electrode, indicating that the operating conditions employed did not promote detectable morphological changes. These results demonstrate the good morphological stability of the electrode during electrolysis and support suitability of 304 stainless steel for repeated applications in the proposed electrochemical reactor. This observation is compared to that reported by Bakhramov and Zhang (2026),^32^ who estimated a lifetime exceeding 200 years for benchmark unmodified 316L stainless steel electrodes for alkaline electrolysis systems over 1000 h test validation.

**Figure 7:**
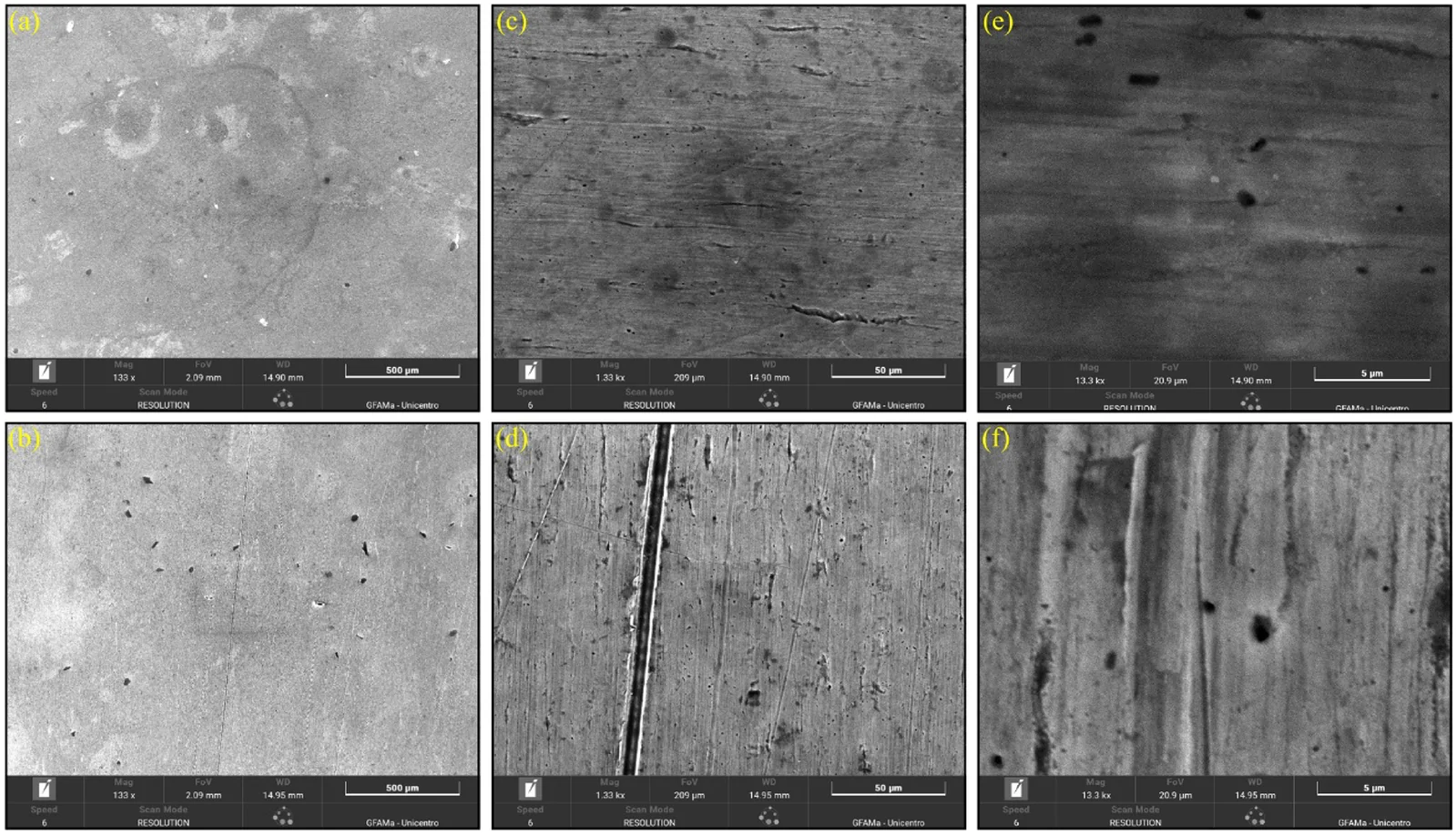
Electrodes surface morphology; (a), (c) and (e) electrodes before electrolysis; (b), (d) and (f): electrodes after electrolysis.

### 3.9 Energy Consumption Analysis

The SEC of the proposed biodiesel production system was evaluated to assess its energy efficiency. Under optimized conditions, the total SEC was 7.20 × 10^–3^ kWh kg^–1^ of biodiesel for an average current of 30, 25 mA in the electrolysis process. This value accounts for the energy demanded by electrolysis and the stirring system. These observations are significantly lower than those reported in the literature for conventional and intensified biodiesel production processes, as reported by Oladipo et al. (2024),^33^ who found an energy consumption of 0.91 kWh kg^–1^ for microwave-assisted biodiesel production. Moreover, the values are lower than those reported by similar studies that produced biodiesel using electrochemical process, as shown by Söyler et al. (2026)^15^ and Khan et al. (2026),^16^ who reported SEC values of 0.25–1.19 and 1.22 kWh kg^–1^, respectively.

The remarkably low SEC observed in this study is attributed to the synergistic combination of the chemical catalysis by KOH and the electrochemical generation of methoxide ions at a low current. This dual mechanism enables high biodiesel conversion while requiring minimal electrical energy input.

## 4 Conclusion

The present study demonstrated the technical feasibility of biodiesel production through an electrochemical reactor powered by photovoltaic energy, using sunflower oil as feedstock and 304 stainless steel electrodes as the working material. The system exhibited operational stability and no visible electrode corrosion, achieving yields above 90% and ester contents higher than 96.5% under optimized conditions, and low energy consumption. A significant reduction in acid value and free fatty acid content was observed in comparison with the initial oil, resulting in a product compatible with the specifications established by ANP and EN 14214 standards.

The intermediate potential of 20 V associated with higher molar ratios (6:1–9:1) proved to be more favorable for the conversion of triglycerides into methyl esters, minimizing secondary hydrolysis reactions. These results indicate that the integration of electrochemical reactor and solar photovoltaic energy represents a promising and environmentally sustainable alternative for biodiesel production, especially in decentralized and small-scale systems. Future studies may address the overall energy efficiency of the process, electrode durability, and the application of the proposed electrochemical reactor to waste cooking oil and other non-edible feedstocks, and a comparative evaluation of alternative electrode materials with respect to catalytic performance, durability, and cost.

## Supplementary Material



## Notes

^1^H NMR spectra of all biodiesel samples (Figures S1–S15) (PDF)
