# Supplementary material for: Development of an Electrochemical Reactor Prototype for Biodiesel Production Powered by Photovoltaic Energy
Source: ACS Omega. 2026 Sep 11;11(37):56314–22. doi: 10.1021/acsomega.6c07137 (PMC13613903; doi:10.1021/acsomega.6c07137)
Supplement: Supplementary file 1 [file ao6c07137_si_001.pdf]

SUPPORTING INFORMATION

DEVELOPMENT OF AN ELECTROCHEMICAL REACTOR PROTOTYPE FOR BIODIESEL PRODUCTION POWERED BY PHOTOVOLTAIC ENERGY

Gabriel Felipe Rohling[a], Josiane de Almeida Freire[a], Katharine Margaritha Satiro Braz[b], Fernanda Oliveira Lima[a] ,André Lazarin Gallina[c] ,Thiago Anjos[d], Ricardo F. Schumacher[d], Letiére Cabreira Soares[a]*

[a]Universidade Federal da Fronteira Sul, *Campus* Realeza, Paraná, Brazil
[b]Universidade de São Paulo, *Campus* Butantã, São Paulo, Brazil
[c]Universidade Estadual do Centro Oeste, *Campus* Cedeteg, Paraná, Brazil
[d]Universidade Federal de Santa Maria, Santa Maria, Rio Grande do Sul, Brazil
***Corresponding author:** letiere.soares@uffs.edu.br

Nuclear Magnetic Resonance (^1H NMR) Spectroscopy

The ^1H NMR spectra were recorded to confirm the conversion of triglycerides into fatty acid methyl esters (FAME). The chemical shifts (δ) are reported in ppm, referenced to the internal standard tetramethylsilane (TMS).

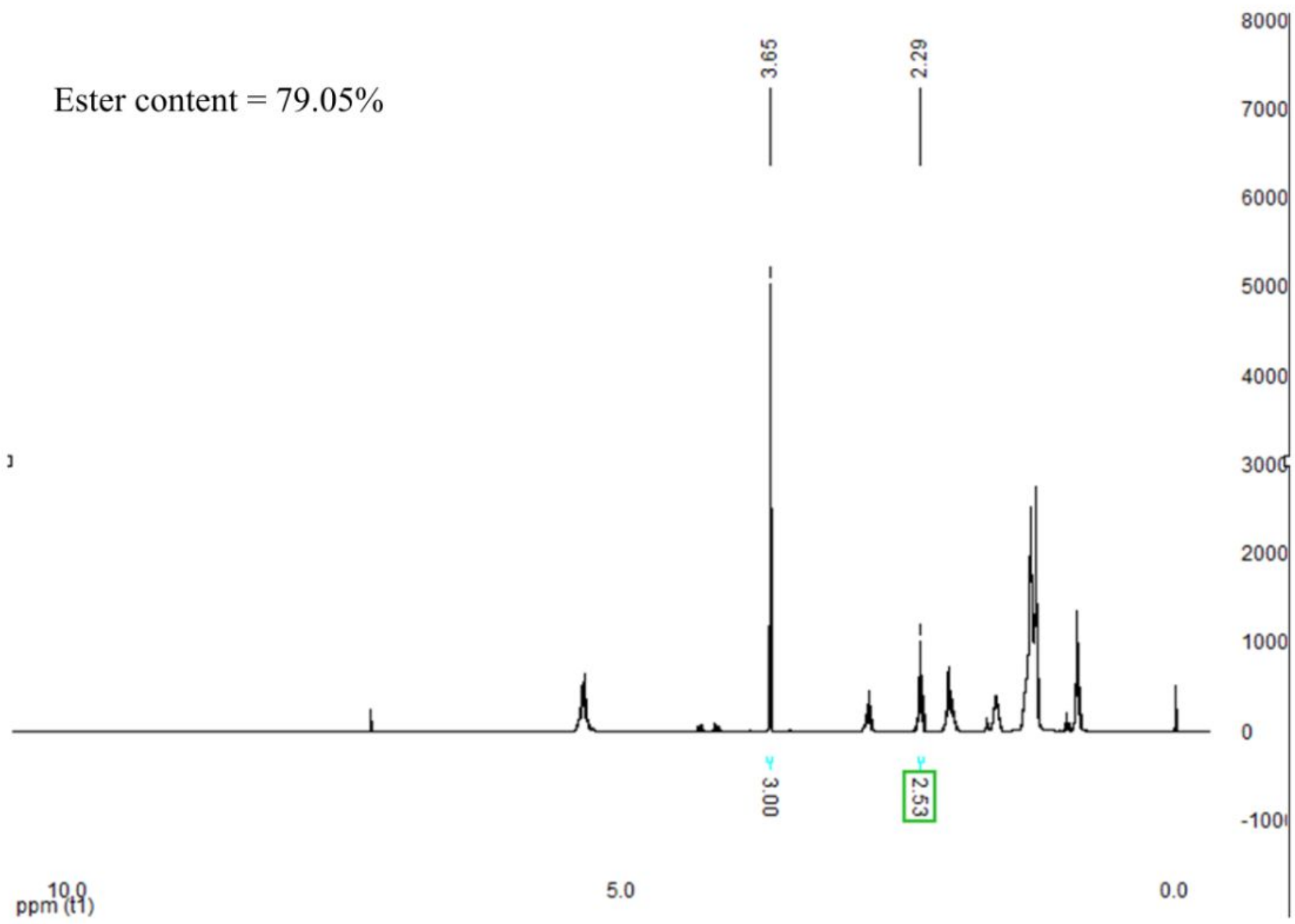

Fig S1. Biodiesel NMR spectra with 1:3 O:M ratio and 10V.

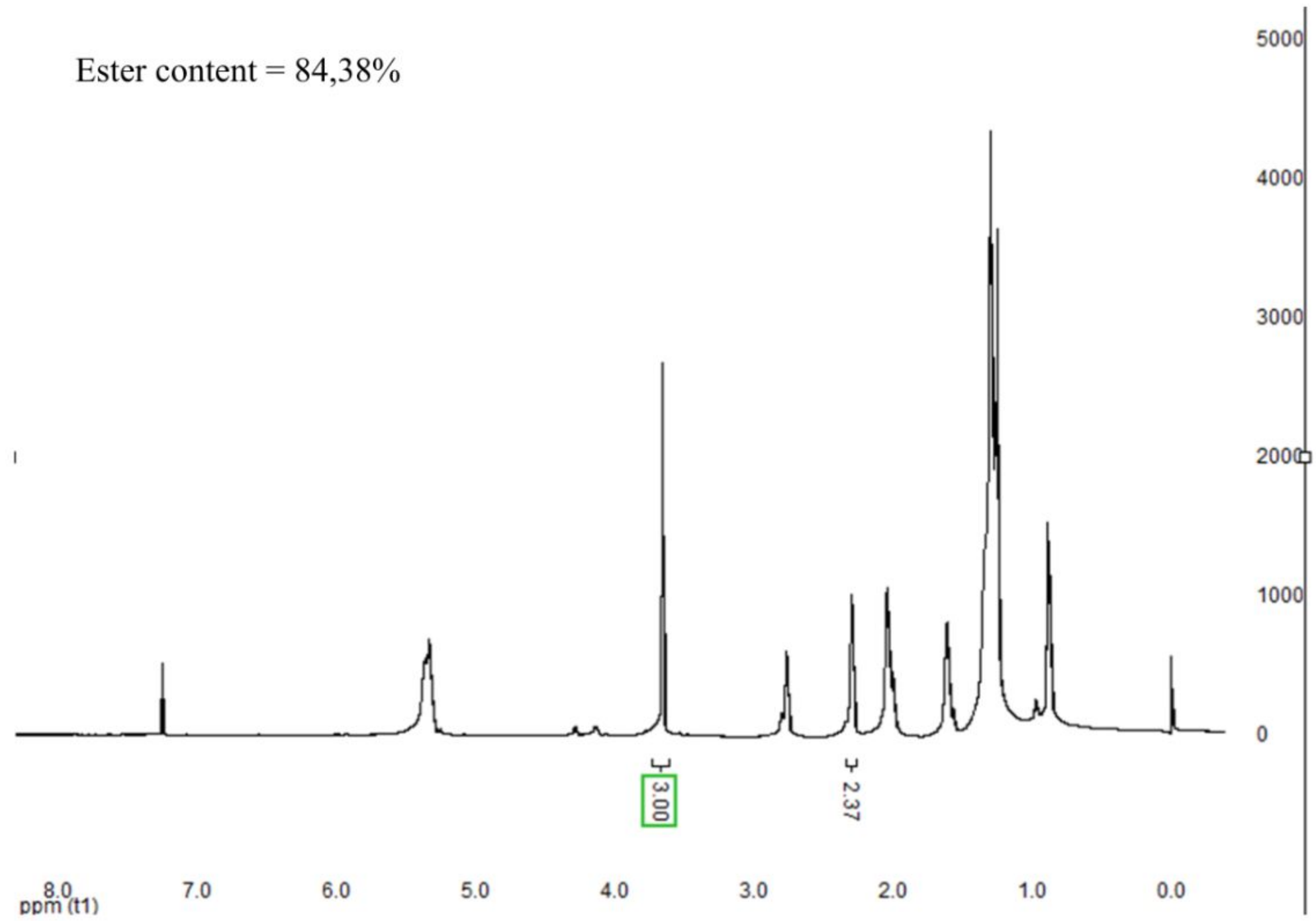

Fig S2. Biodiesel NMR spectra with 1:3 O:M ratio and 20V.

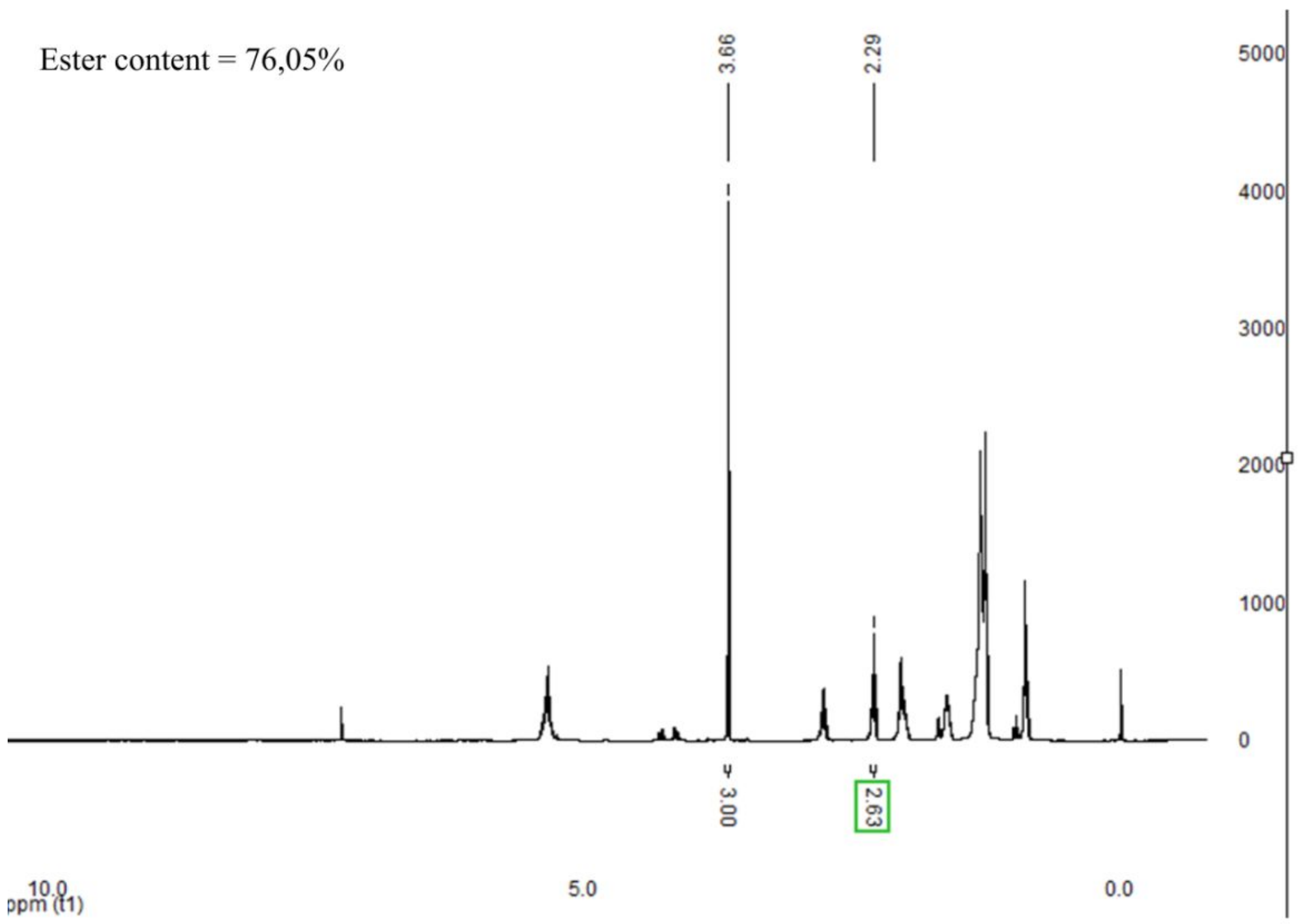

Fig S3. Biodiesel NMR spectra with 1:3 O:M ratio and 30V.

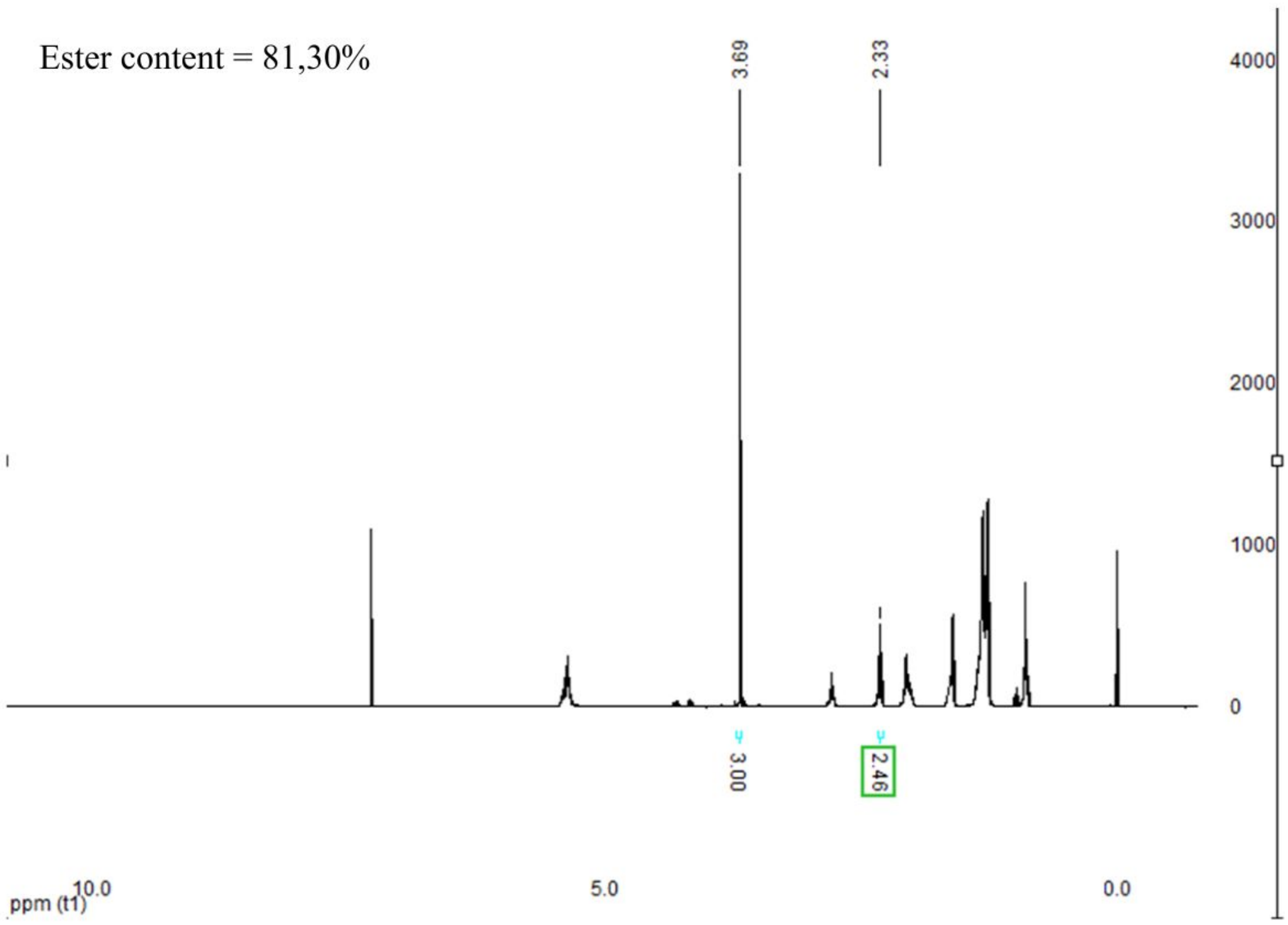

Fig S4. Biodiesel NMR spectra with 1:3.5 O:M ratio and 10V.

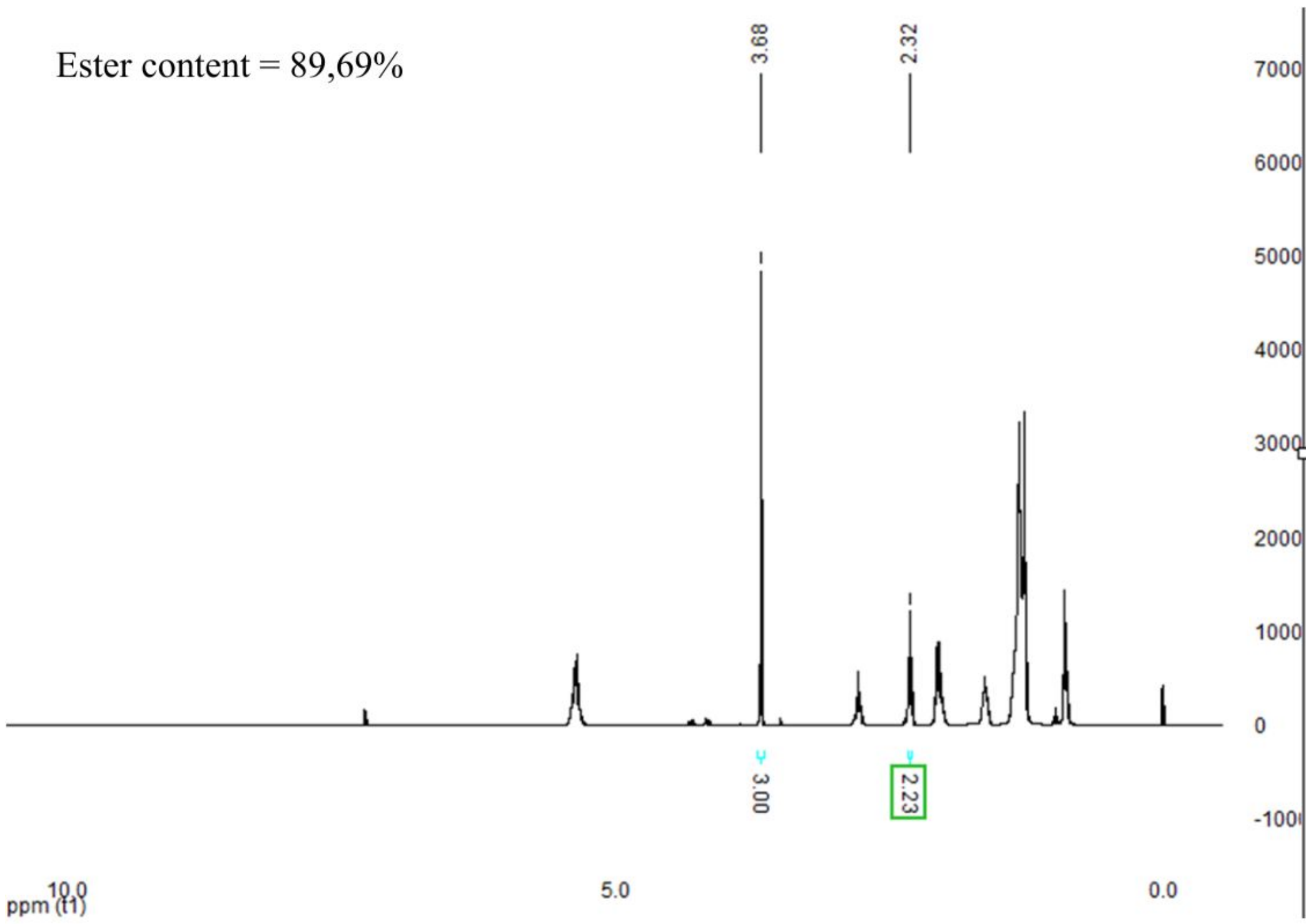

Fig S5. Biodiesel NMR spectra with 1:3.5 O:M ratio and 20V.

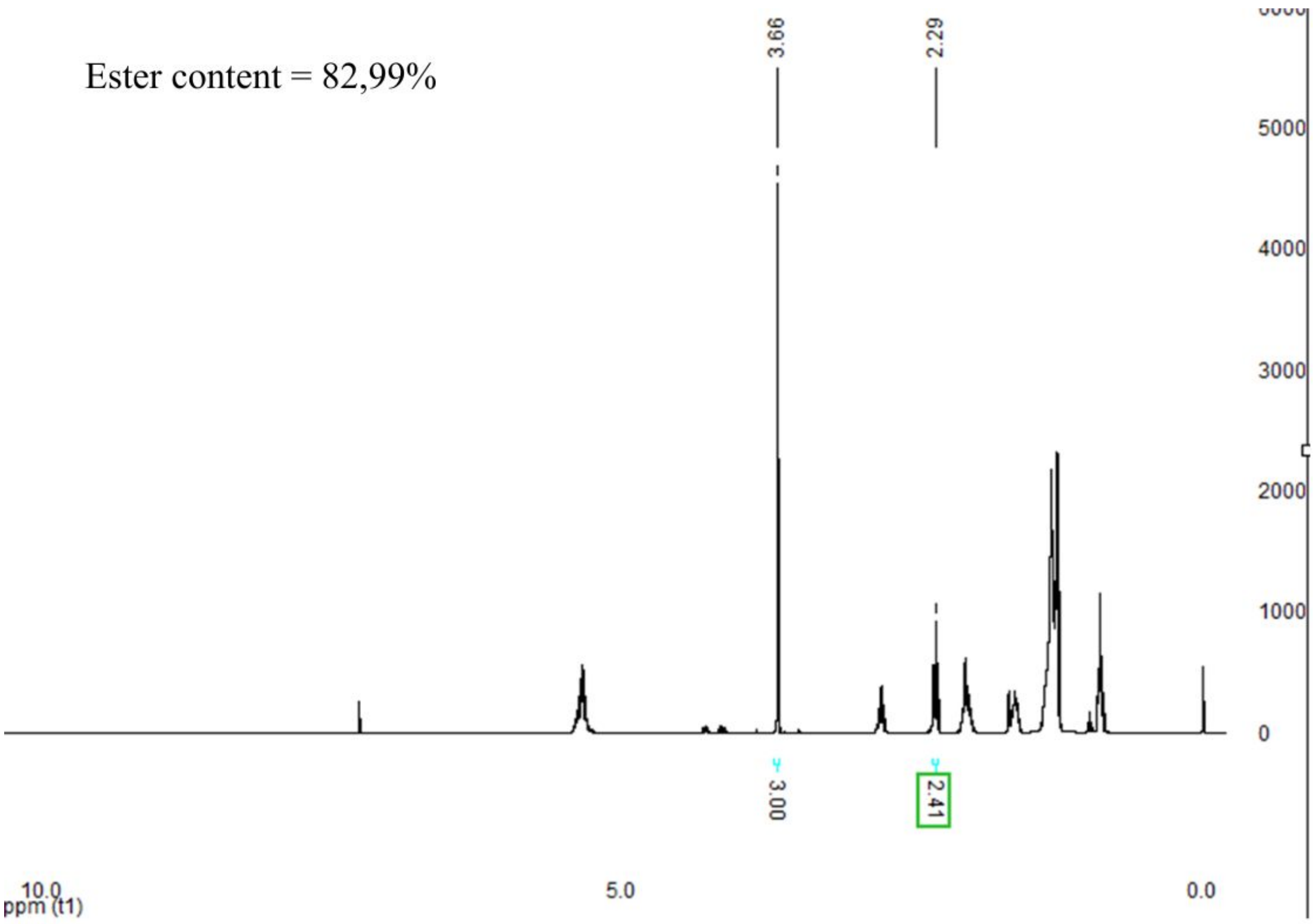

Fig S6. Biodiesel NMR spectra with 1:3.5 O:M ratio and 30V.

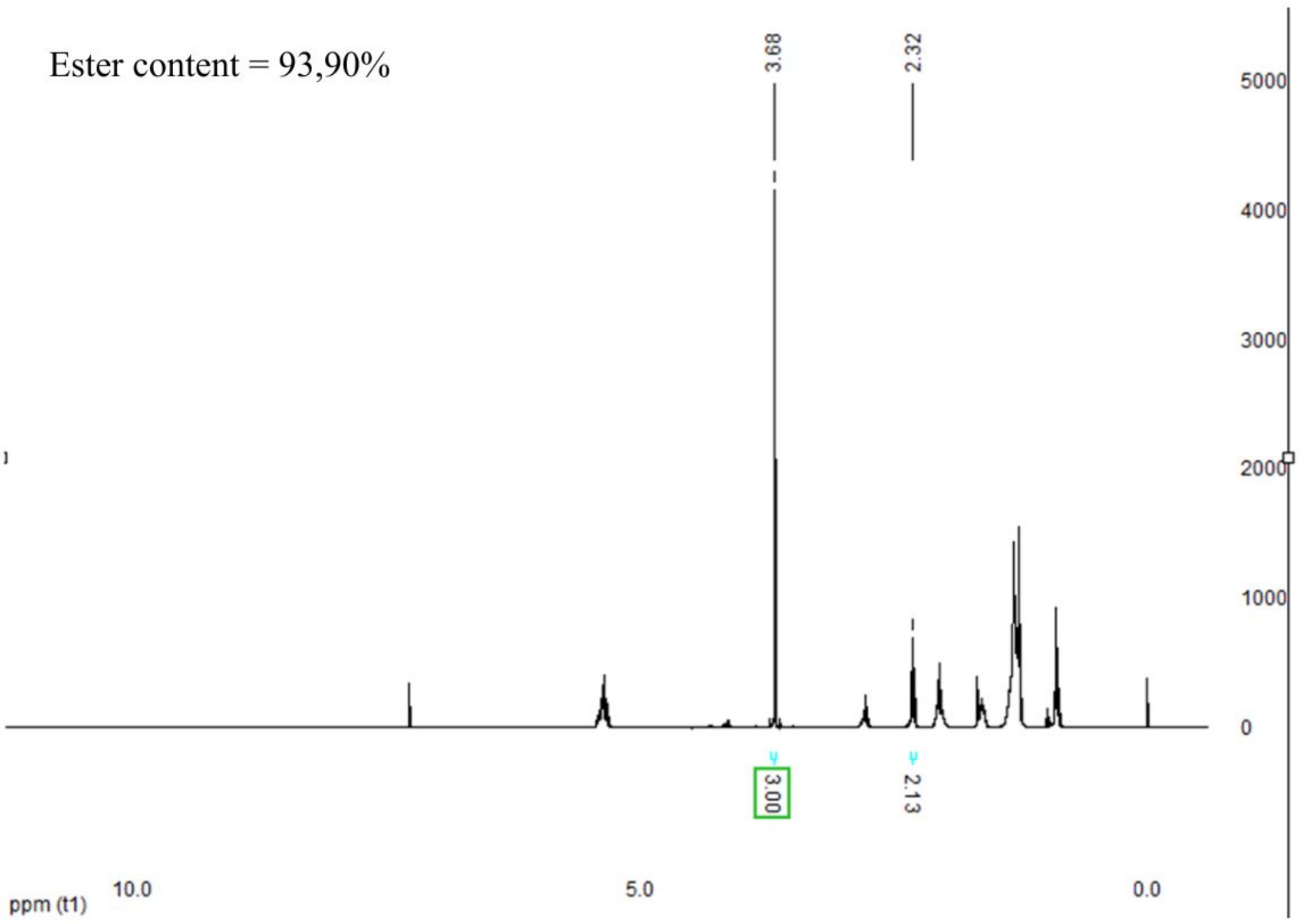

Fig S7. Biodiesel NMR spectra with 1:4 O:M ratio and 10V.

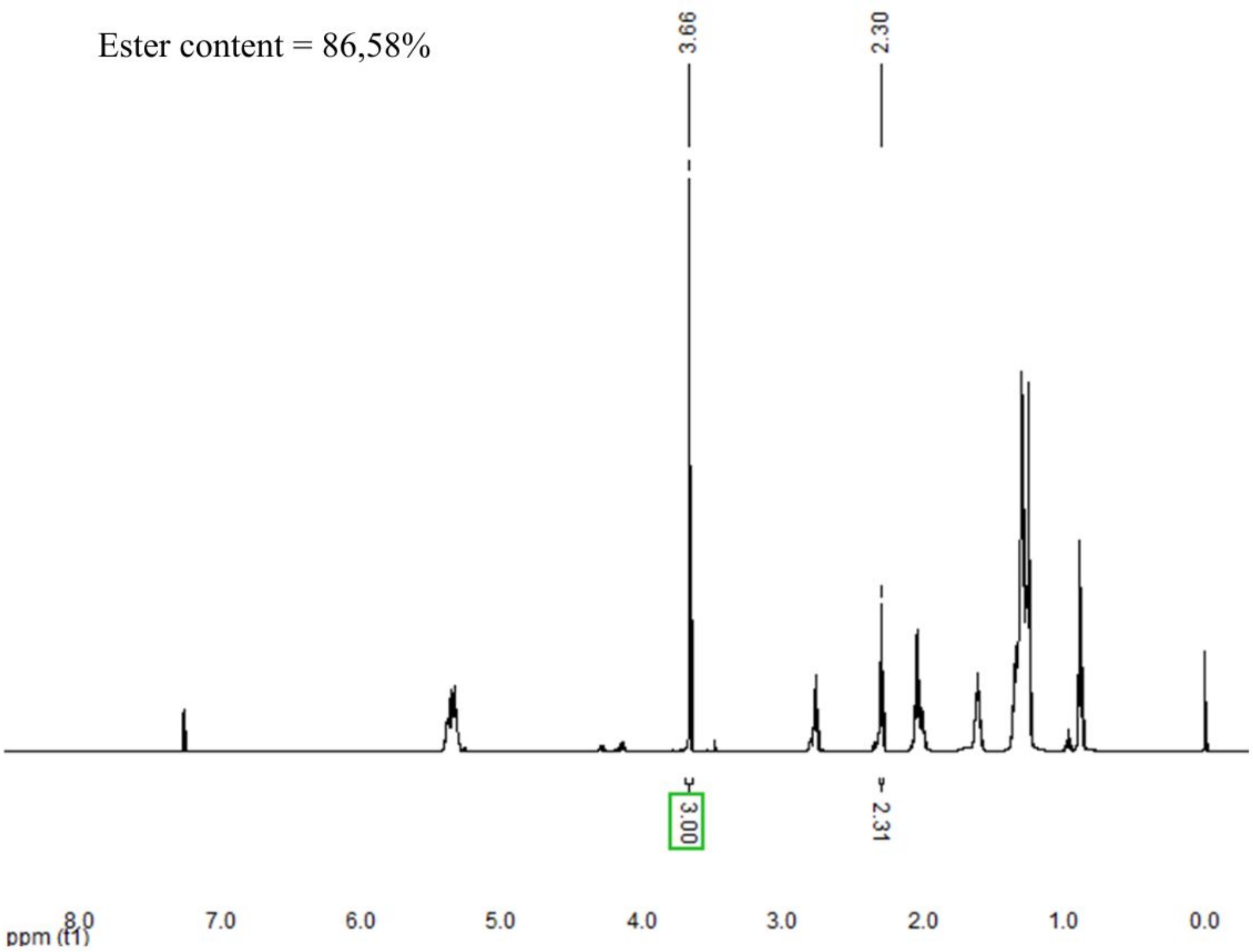

Fig S8. Biodiesel NMR spectra with 1:4 O:M ratio and 20V.

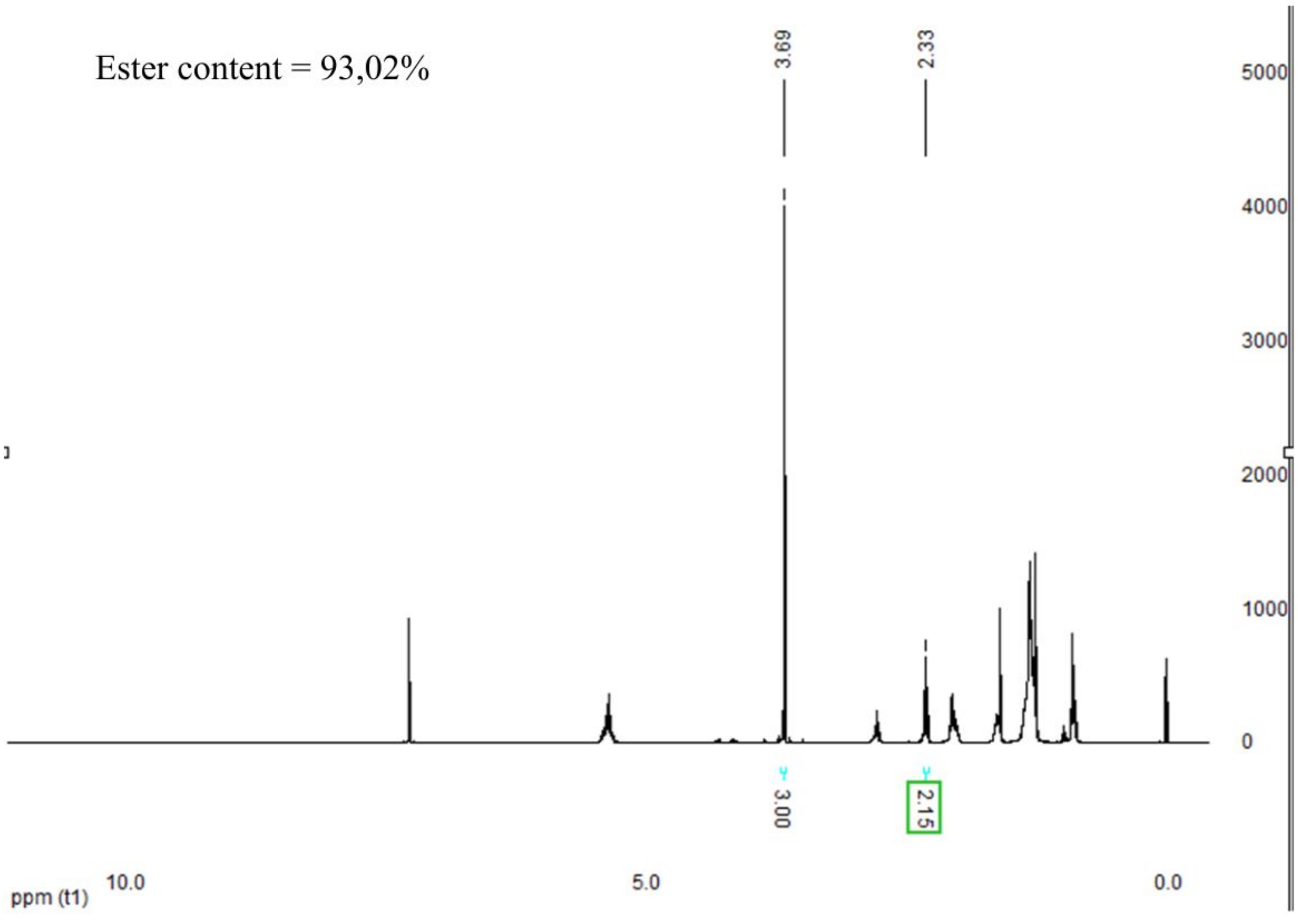

Fig S9. Biodiesel NMR spectra with 1:4 O:M ratio and 30V.

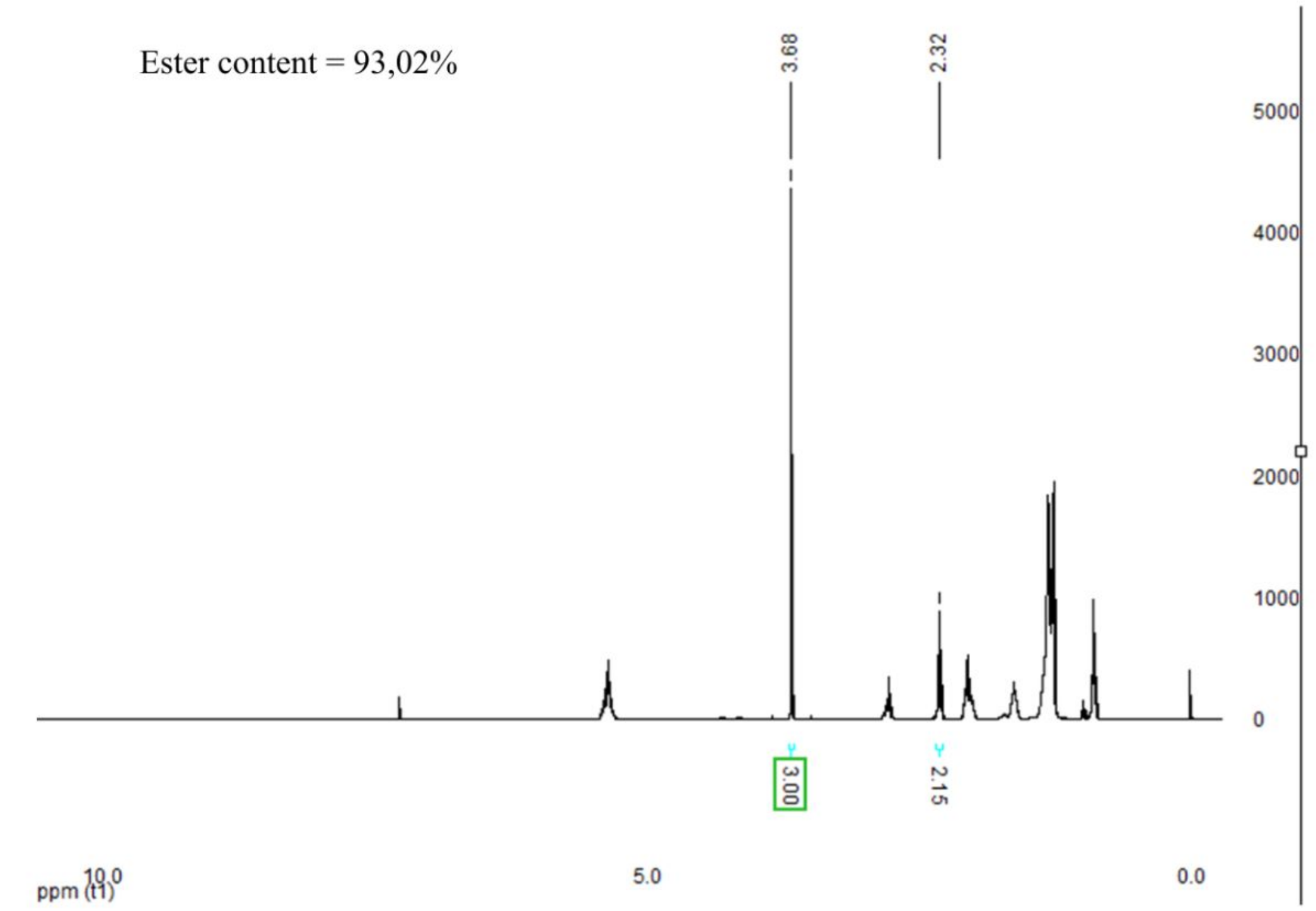

Fig S10. Biodiesel NMR spectra with 1:6 O:M ratio and 10V.

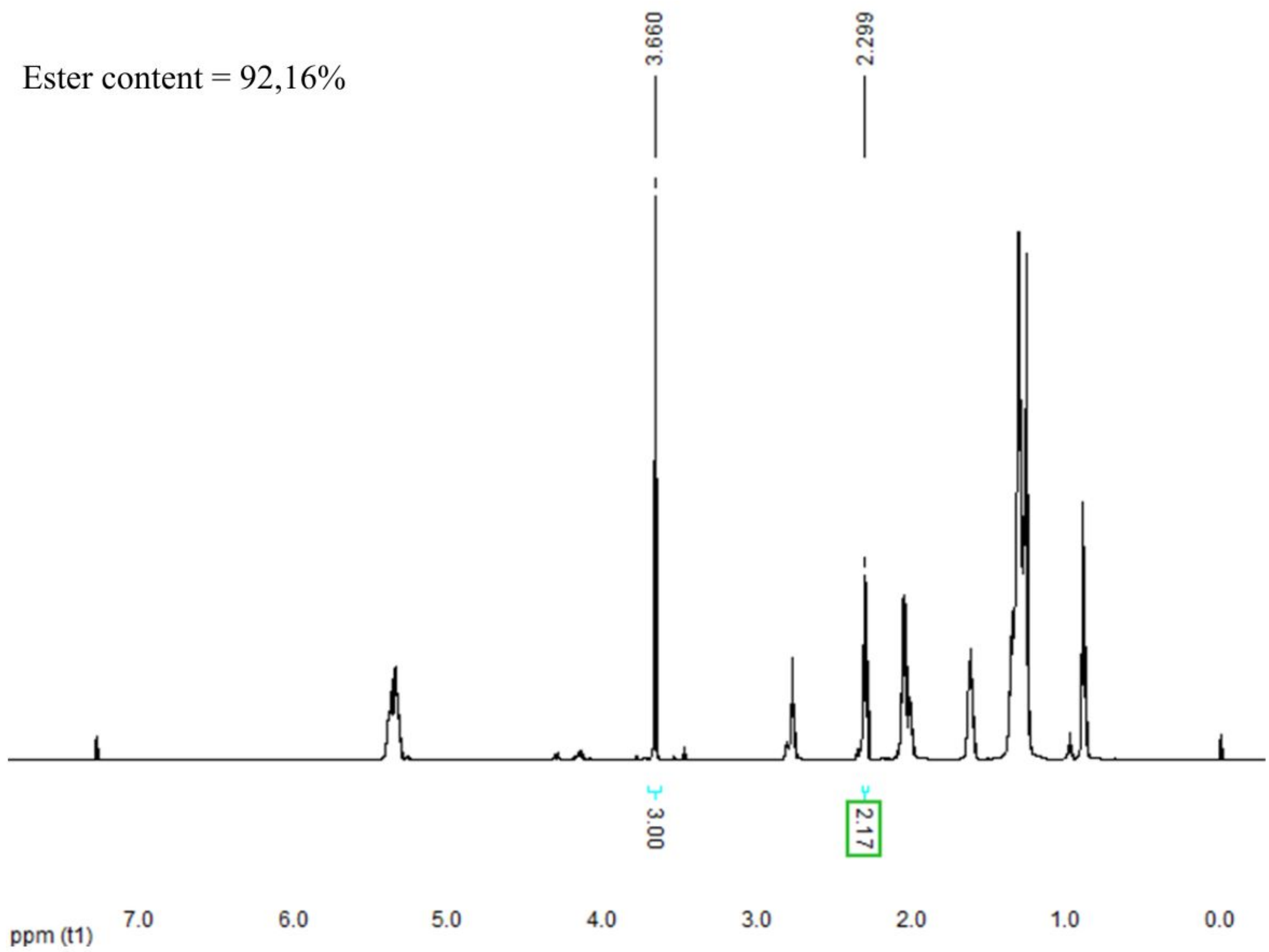

Fig S11. Biodiesel NMR spectra with 1:6 O:M ratio and 20V.

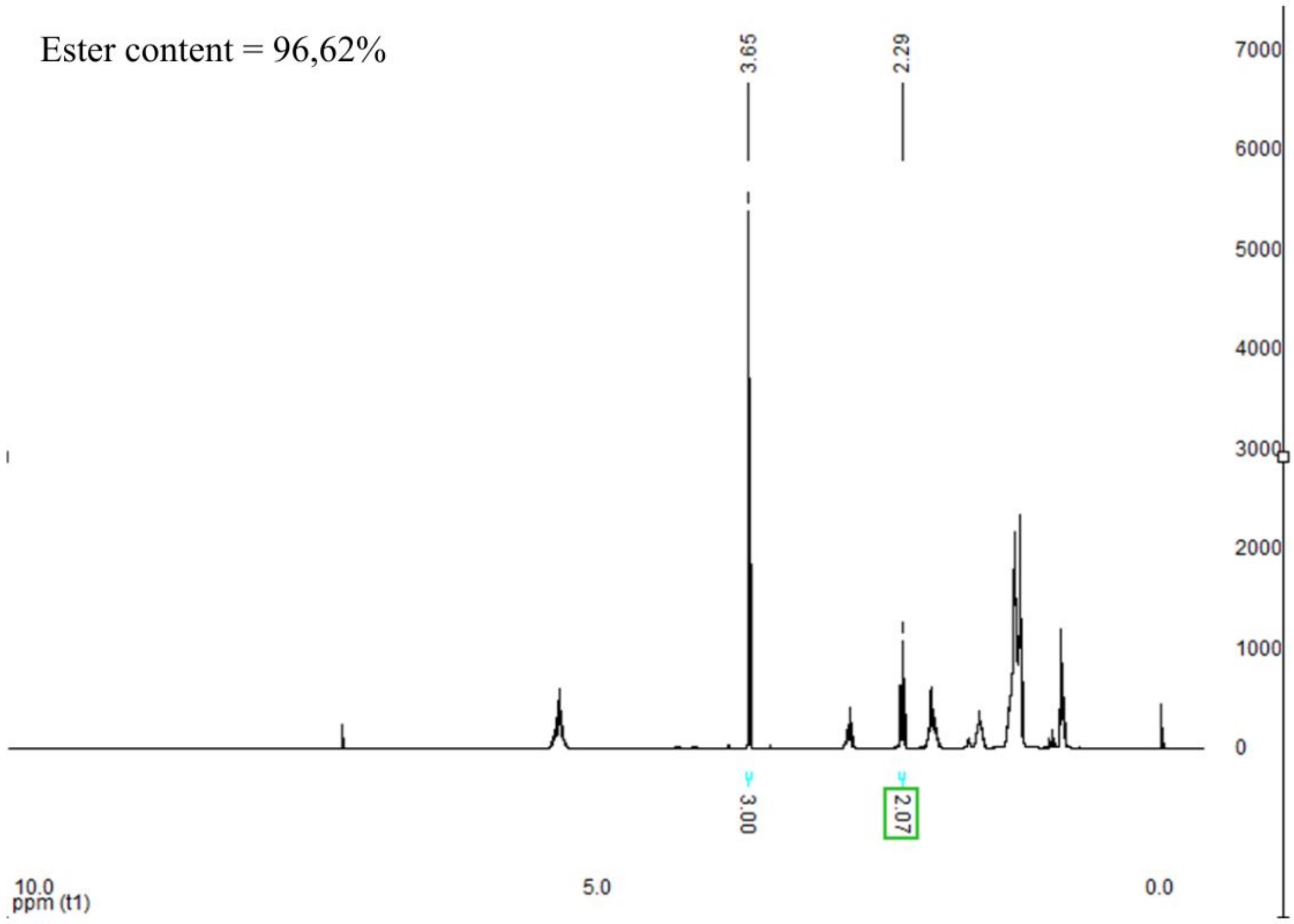

Fig S12. Biodiesel NMR spectra with 1:6 O:M ratio and 30V.

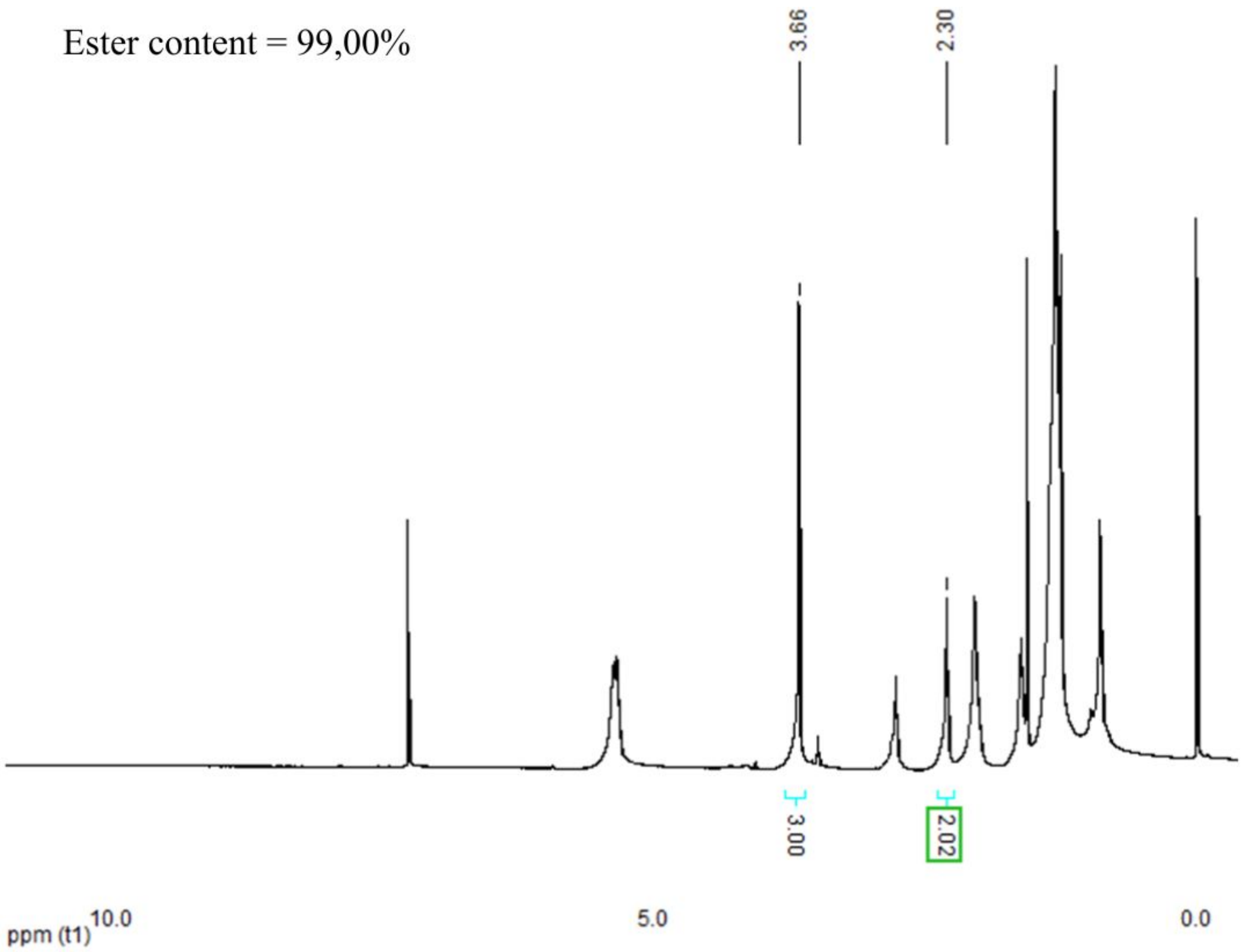

Fig S13. Biodiesel NMR spectra with 1:7 O:M ratio and 10V.

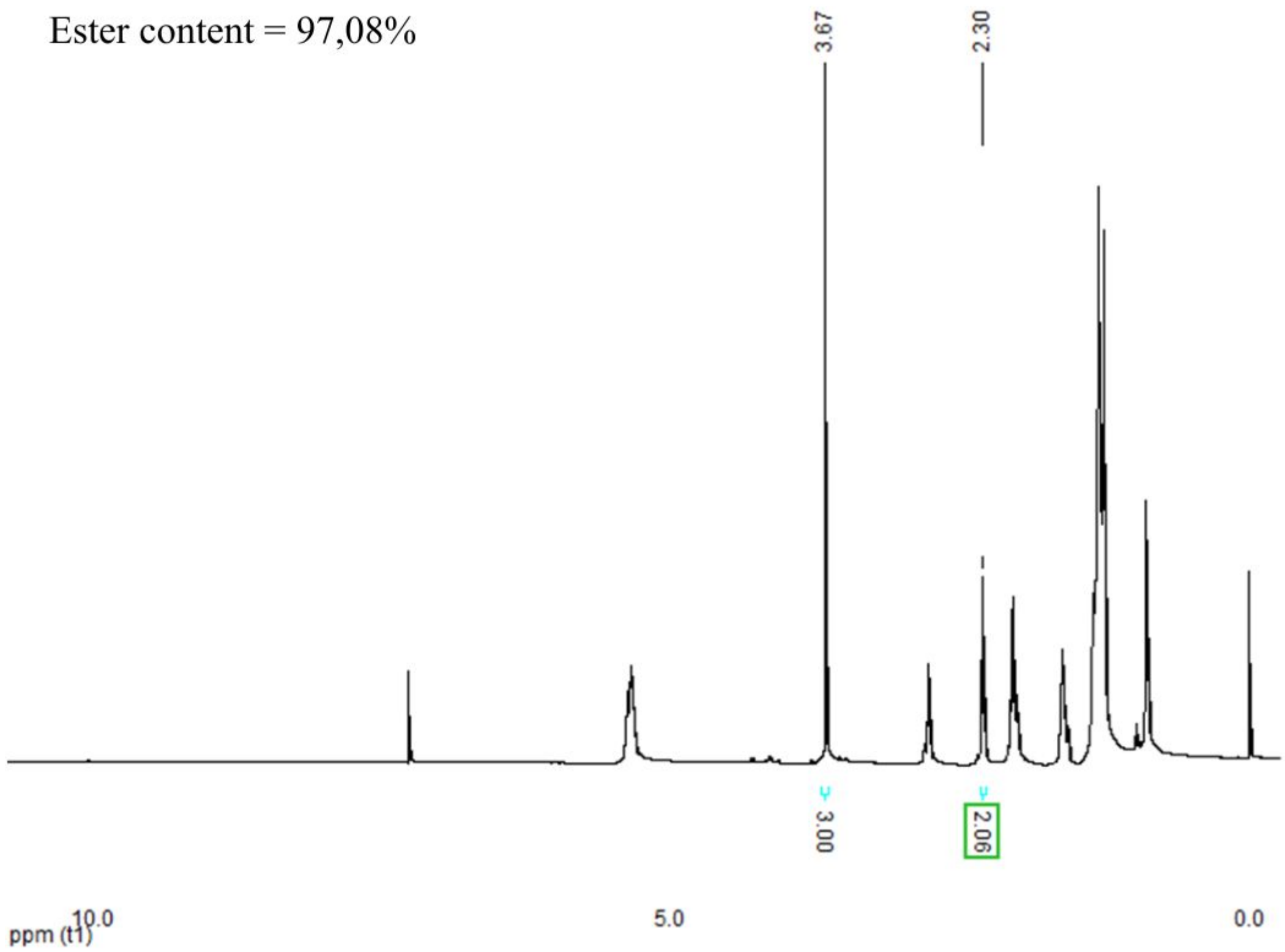

Fig S14. Biodiesel NMR spectra with 1:7 O:M ratio and 20V.

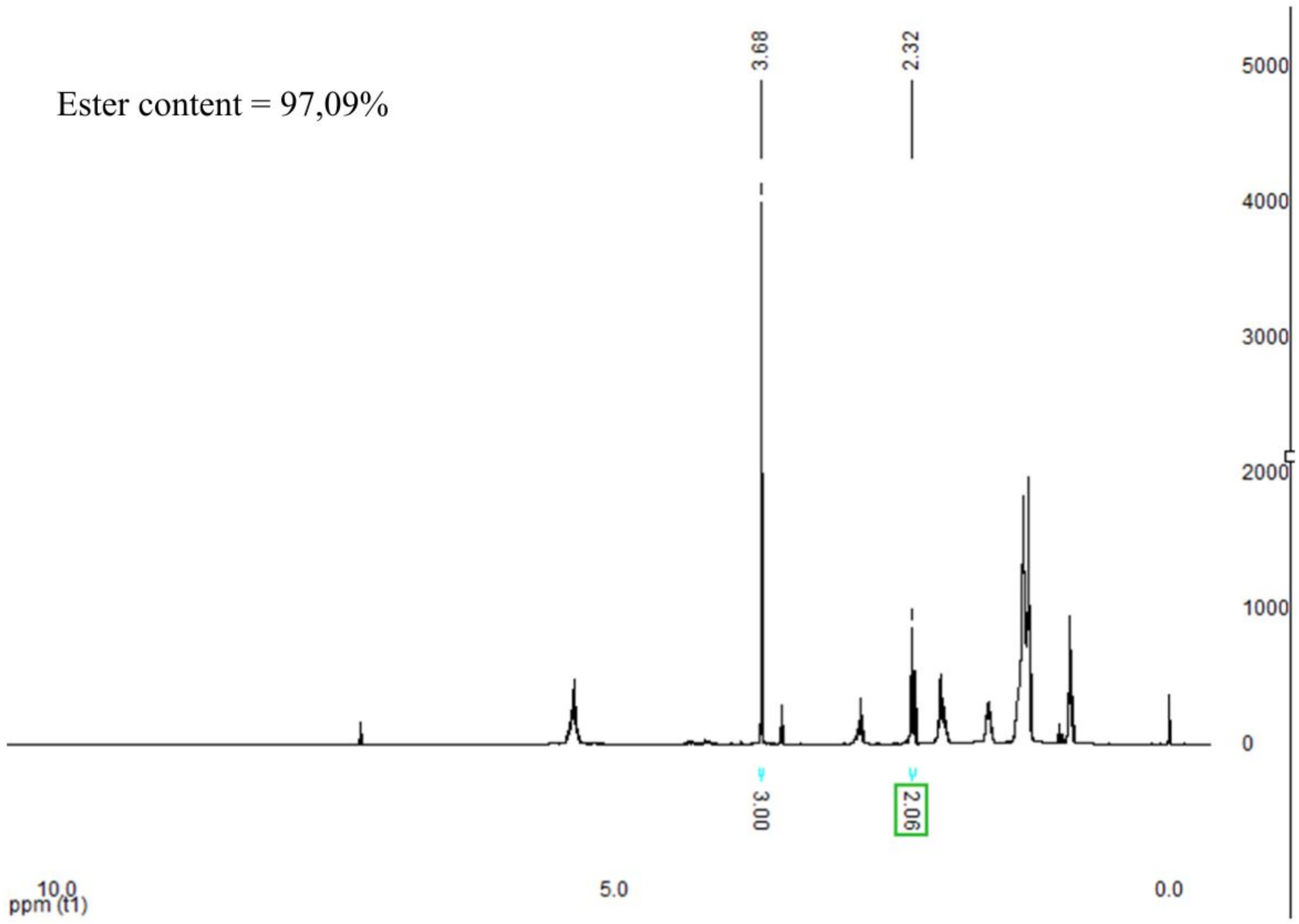

Fig S15. Biodiesel NMR spectra with 1:7 O:M ratio and 30V.
